# Complete Mitochondrial Genomic Characteristics and Phylogenetic Analysis of the Medicinal Plant *Peperomia leptostachya*

**DOI:** 10.3390/genes17010118

**Published:** 2026-01-22

**Authors:** Mengyun Ying, Jianyu Shi, Zhijun Shen, Qiuping Ye

**Affiliations:** Fujian Institute of Subtropical Botany, Fujian Key Laboratory of Subtropical Plant Physiology and Biochemistry, Xiamen 361006, China; yingmengyun1989@126.com (M.Y.); shenzj1987@xmu.edu.cn (Z.S.); qiupingye@126.com (Q.Y.)

**Keywords:** *Peperomia leptostachya*, mitochondrial genome, repeat sequences, RNA editing, phylogenetic relationships

## Abstract

**Background**: *Peperomia leptostachya* is a herbaceous plant with significant medicinal value. To elucidate its mitochondrial genomic characteristics, this study conducted a systematic analysis. **Methods**: The mitochondrial genome of *P. leptostachya* was assembled, annotated, and subjected to comparative analysis. **Results**: (1) The genome exhibits significant structural peculiarities, presenting as an atypical circular structure accompanied by an independent minicircle, forming a multi-branched reticulate configuration spanning a total length of 981,249 bp. Within the mitochondrial genome of *P. leptostachya*, a total of 52 genes have been identified, including 35 PCGs, 14 tRNAs and 3 rRNAs. (2) A phylogenetic tree was built for 22 species based on the DNA sequences. *P. leptostachya* belongs to the family Piperaceae within the order Piperales and is closely related to *Piper nigrum*. (3) Homologous colinear blocks were detected between *P. leptostachya* and its close relatives, though these blocks exhibited short lengths. Additionally, blank regions were identified that showed no homology with other species. Mitochondrial genomes of *P. leptostachya* and two close relatives had inconsistent collinear block arrangements. The mitochondrial genome of *P. leptostachya* had undergone genomic rearrangement relative to closely related species. **Conclusions**: This study lays the foundation for research into the genetic characteristics and biological traits of *P. leptostachya*.

## 1. Introduction

*Peperomia leptostachya*, belonging to the genus Peperomia in the Piperaceae family, is an annual fleshy herb, born in valleys, streamsides or under forests in stone crevices and on moist rocks, distributed in southern provinces and districts of China [1,2]. It is primarily used to treat conditions such as cough, asthma, stomach cancer, and lung cancer [3,4]. This species has attracted much attention due to its unique secondary metabolites. *P. leptostachya* mainly contains chemical components such as lignans, flavonoids, and polyketides, among which the lignan content is higher [5,6,7,8].

At present, domestic and international studies on the activity of *P. leptostachya* are mostly focused on medical aspects [3,9,10,11,12]. However, little has been reported on species identification, phylogenetic analysis and genetic studies. At present, genomic studies of Piperaceae are mainly focused on chloroplast genomes, while mitochondrial genomes have not yet been systematically resolved due to highly repetitive sequences and complex recombination problems [13]. The complete mitochondrial genomes of Piperaceae are relatively few.

Recent studies on the mitochondrial genomes of multiple species have revealed that their unique structural features—such as polyploidy and abundant repetitive sequences—along with dynamic processes like RNA editing and the transfer of chloroplast-derived DNA fragments, are key to understanding species evolution and adaptive mechanisms [14,15,16]. In plants, the mitochondrial genetic code does not differ from the universal genetic code. The study of the mitochondrial genome, large-scale phylogenetic analyses, and species classification can make the study of genetic variation and species adaptation more systematic and comprehensive [17] and can provide a deeper understanding of the ecological adaptive mechanisms of biological groups. Genomic approaches supported by molecular biology and bioinformatics are of critical importance to document and conserve biodiversity [18]. Plant mitogenomes can be circular, linear, branched, multichromosomal, or a combination of structural forms as verified by fluorescence microscopy. At present, it is apparent that the real configurations of most land plant mitogenomes vary greatly via recombination mediated by repeats [19]. The primary characteristics of plant mitochondrial genomes are: enormous variation in both size and structure; genes that are yet extremely conserved; and extremely sparse gene distribution. The very low mutation rates in land plant mtDNA create an environment where noncoding DNA and other genomic changes can accumulate more easily; consequently, extra DNA tends to accumulate in genomes with low mutation rates, which helps explain why plant mtDNA has low overall mutation rates but large genome sizes [20]. RNA editing influences key post-transcriptional regulatory mechanisms in mitochondria, affecting mitochondrial protein synthesis and function by altering transcript sequences. The fast evolutionary rate of the mitochondrial genome and the diverse molecular markers it offers enable it to be an effective means for investigating system evolution [21].

The mitochondrial genome provides a unique molecular tool for medicinal plant research, offering crucial evidence for resolving the phylogenetic relationships of complex medicinal plant taxa. Simultaneously, the composition of the mitochondrial genome is considered fundamental to understanding energy metabolism and environmental adaptation in plants [22]. RNA editing influences post-transcriptional regulation of mitochondrial proteins and plant traits, affecting plant energy metabolism and thereby indirectly regulating the synthesis of secondary metabolites. In medicinal plant research, the analysis of mitochondrial genomes not only aids in elucidating species taxonomic status (such as confirming the phylogenetic relationship between Punica granatum and Lagerstroemia indica in phylogenetic analyses) [15], but also reveals the potential genetic basis for important traits such as cytoplasmic male sterility (CMS) [14]. This functional-level regulation, combined with genomic structural features, enables a more systematic establishment of scientific connections between mitochondrial genomic characteristics and species ecological adaptability.

Based on the above, we hypothesize that the mitochondrial genome of *P. leptostachya* exhibits a unique and complex structure. Furthermore, we propose that its dynamic genomic features—such as repetitive sequences and RNA editing—are associated with adaptive and post-transcriptional regulatory mechanisms. The present study aims to address the following questions: (1) What is the structure of its mitochondrial genome? What is the phylogenetic position and relationship of this species within the relevant taxonomic group? (2) What patterns and biological significance do the abundant RNA editing events exhibit? (3) To what extent do mitochondrial plastid DNAs (MTPTs) retain functional integrity?

This study presents the first systematic analysis of the *P. leptostachya* mitochondrial genome, revealing its complex structural morphology, extensive genomic rearrangements, abundant repetitive sequences, and frequent RNA editing events. The mitochondrial genome of *P. leptostachya* was assembled, annotated, and subjected to comparative analysis. Furthermore, we explored the genetic characteristics, evolutionary history, and ecological adaptations of *P. leptostachya* from the organelle genome functional level. A phylogenetic tree was constructed, and comparative analysis of synteny patterns elucidated genomic structural variations among closely related species. Therefore, this study not only provides foundational insights into *P. leptostachya* but also offers a reference for exploring the characteristic structures and phylogenetic relationships of Piperaceae plants. Furthermore, it presents a new research case for understanding the characteristics of plant mitochondrial genomes.

## 2. Materials and Methods

### 2.1. Plant Materials and Sequencing

The plants used in this experiment were collected from Wudi Village, Gongqiao Town, Zhangping City, Fujian Province, China (25°17′24″ N, 117°24′36″ E). They were identified by Senior Agronomist Chen Hengbin of Xiamen Botanical Garden as *P. leptostachya* (Piperaceae family, genus Peperomia) and cultivated in the medicinal plant greenhouse of Fujian Subtropical Botanical Garden. In March 2024, we harvested 50 apical cuttings (10 cm long) and propagated them in five planting baskets (40 cm × 30 cm × 20 cm each, 10 cuttings per basket) using a growing medium of red soil: peat moss = 1:3. By July 2024, one plant exhibiting uniform growth was selected from each tray. The third leaf from the top was collected as a sample, yielding five replicate samples. Samples were immediately flash-frozen in liquid nitrogen and stored at −80 °C in an ultra-low temperature freezer (Zhejiang Heli Refrigeration Equipment Co., Ltd., Jinhua, China). Mixed tissue samples were stored for one week before testing. From the foliage, employing the method known as Cetytrimethylammonium Bromide (CTAB), total genomic DNA was extracted [16], and the extracted DNA was detected and subjected to high-throughput sequencing to construct a *P. leptostachya* gene library. The libraries were sequenced using the Illumina NovaSeq 6000 platform (Illumina, San Diego, CA, USA) for second-generation sequencing and the ONT sequencing platform for third-generation single-molecule sequencing. The ONT sequencing data size was 11.86 Gb, comprising 1,002,263 reads. The longest read was 366,499 bp, with an average read length of 11,833.2 bp. The Q-score was 15.07. Sequencing and data analysis services were provided by Wuhan GeneRead Biotechnology Co., Ltd. (Wuhan, China).

### 2.2. Mitochondrial Genome Assembly and Annotati

With respect to the acquisition of graphical assembly outputs in GFA format, direct assembly was implemented on long-read sequencing data utilizing Flye’s default parametres [23]. During the initial long-read assembly with Flye, the read length distribution showed an N50 of 14,733 bp and an N90 of 7381 bp. Accordingly, the minimum overlap parameter was set to 7000 bp. Employing makeblastdb, the library was constructed for all contigs presented in the fasta format; subsequent employment of the BLASTn program facilitated the identification of those contig fragments that encompassed segments of the mitochondrial genome. The mitochondrial genes inherent to Arabidopsis thaliana functioned as the query sequence within this endeavor. The assembly results of *P. leptostachya* were visualized and examined with Bandage (v0.8.1) [24]. Long-read and short-read data were aligned to contigs with BWA (v0.7.17) [25], with filtered mitochondrial reads exported from the comparison. Mixed assembly was achieved with Unicycler [26] to finalize the *P. leptostachya* mitochondrial genome. Using the assembled mt contigs as a reference sequence, long reads from the raw sequencing data were mapped to the reference. Results with mapped lengths exceeding 5000 bp were retained, with a total of 3117 long reads successfully mapped. Coverage maps for the mt contigs were generated, showing an average coverage depth of 41.8× for contig1 and 37.6× for contig2.

Through employing the reference genomes of both Liriodendron tulipifera (NC_021152.1) and Arabidopsis thaliana (NC_037304), an annotation of protein-coding genes was conducted, wherein tools such as Geseq (v2.03) [27], alongside PMGA [28] for angiosperm mitochondrial genome annotation, were utilized. We annotated the assembled mitochondrial genome using PMGA. PMGA invokes the MAKER tool for annotation based on 319 conserved plant mitochondrial coding genes. Protein-coding genes with similarity retention above 85% were retained. Geseq software (v2.03) served as the reference. Results showed that the number of annotated genes in Geseq matched PMGA, but discrepancies existed in the lengths and positions of specific coding sequences. We retained PMGA’s results because its database is more accurate and comprehensive. The annotated sequences were verified to possess start codons and stop codons conforming to gene rules, with no internal premature stop codons. tRNA and rRNA annotations employed tRNAscan-SE (v.2.0.11) [29] and BLASTN (v2.13.0) [30], respectively.

### 2.3. Analysis of RSCU

Codon bias assessment for mitochondrial gene-encoded proteins was conducted via Phylosuite (v1.1.16) [31] and Mega (v7.0) [32]. The calculated RSCU values reveal these preferential characteristics.

### 2.4. Repeat Sequences Analysis

Repeat sequences were detected with MISA (version2.1) [33], TRF (version4.09) [34], and REPuter web servers [35]. When performing repetitive sequence analysis using REPuter, the length threshold was set to no less than 50 bp. Data visualization employed Circos (v0.69.9) [36].

### 2.5. Analysis of Mitochondrial Genome Structure

The raw long-read sequences underwent assembly with Flye’s default parameters [24]. To tackle the repeat area within the assembled genome, we employed bwa [26] to align mitochondrial long reads against these repetitive sequences, thereby identifying the pathways best supported by read evidence to resolve ambiguities. This same approach was applied to remaining contigs featuring multiple linkers to facilitate proper resolution. Additionally, we worked to deduce the most plausible genomic architecture of the mitochondrial genome.

### 2.6. Sequence Migration Analysis

We assembled the chloroplast genome employing GetOrganelle [37], followed by annotation with CPGAVAS2 [38], and subsequently polished the annotation outcomes utilizing CPGView [39]. To identify homologous segments, we ran BLASTN (v2.13.0) [30] and presented a visualization of the research findings with Circos (v0.69.9) [36]. BLASTN parameters: -outfmt 6-evalue 1e-5-perc_identity 70. When counting MTPT fragments, we removed redundant data: if different regions of chloroplasts shared homology with the same position in mitochondria, we recorded this as a single transfer event. All MTPTs underwent manual inspection and deduplication.

### 2.7. Phylogenetic Analysis

The phylogenetic analysis included 22 species: *P. leptostachya* (PQ468038.1-PQ468039.1), *Aristolochia fimbriata* (OP649454.1-OP649456.1), *Saururus chinensis* (OQ539548.1-OQ539550.1), *Aristolochia californica* (PV439854.1), *Piper nigrum* (PV439855.1), *Houttuynia cordata* (PQ214805.1-PQ214806.1), *Hernandia nymphaeifolia* (NC_063145.1), *Machilus pauhoi* (OR168698.1-OR168699.1), *Chimonanthus praecox* (OR811177.1), *Caryodaphnopsis henryi* (NC_088584.1), *Cinnamomum camphora* (NC_086632.1), *Cinnamomum chekiangense* (NC_082065.1), *Magnolia officinalis* (NC_064401.1), *Magnolia biondii* (NC_049134.1), *Liriodendron tulipifera* (NC_021152.1), *Magnolia figo* (NC_082234.1), *Magnolia liliiflora* (NC_085212.1), *Chloranthus spicatus* (NC_084267.1), *Hedyosmum orientale* (NC_082064.1), *Sarcandra glabra* (OR668929.1-OR668930.1), *Nymphaea* ‘Joey Tomocik’(NC_060361.1) [40], and *Nymphaea colorata* (NC_037468.1). The gene set employed comprised 33 shared protein-coding genes (*atp1*, *atp4*, *atp6*, *atp8*, *atp9*, *ccmB*, *ccmC*, *ccmFC*, *ccmFN*, *cob*, *cox1*, *cox2*, *cox3*, *matR*, *mttB*, *nad1*, *nad2*, *nad3*, *nad4*, *nad4L*, *nad5*, *nad6*, *nad7*, *nad9*, *rpl5*, *rpl10*, *rpl16*, *rps2*, *rps3*, *rps11*, *rps12*, *rps14*, and *rps19*). By leveraging kinship relationships, we downloaded mitochondrial genomes from phylogenetically close organisms. We employed PhyloSuite (v1.1.16) [31] to identify common genes and employed MAFF (v7.505) [41] for several sequence alignments. Next, we constructed a phylogenetic tree with IQ-TREE (v1.6.12) [42]. The analysis employed the GTR + F + I + R2 model, and node support was assessed with 1000 bootstrap replicates. The phylogenetic findings were depicted with ITOL (v6) [43].

### 2.8. RNA Editing Event Analysis and Collinearity Analysis

The Deepred-mt [44] tool identified C-to-U RNA editing sites within mitochondrial protein-coding genes (PCGs). RNA editing events were identified for 35 unique PCGs from *P. leptostachya* mitochondria. The cutoff value was set to 0.9. This threshold represents the probability value predicted by Deepred-mt for RNA editing events occurring at this site. Deepred-mt employs a CNN-based binary classification model. Predictions are made using input sequences comprising the predicted site and 41 base pairs flanking it on either side. The final output is a probability value ranging from 0 to 1, where higher values indicate a greater likelihood of C-U editing occurring. We retained only probability values above 0.9 to ensure the inclusion of RNA editing sites with high confidence. Using MCscanX [45], pairwise comparisons were generated from the results obtained by the BLASTn program to create multiple synteny plots, mapping conserved syntenic blocks.

## 3. Results

### 3.1. Mitochondrial Genome Structure of P. leptostachya

**Figure 1:**
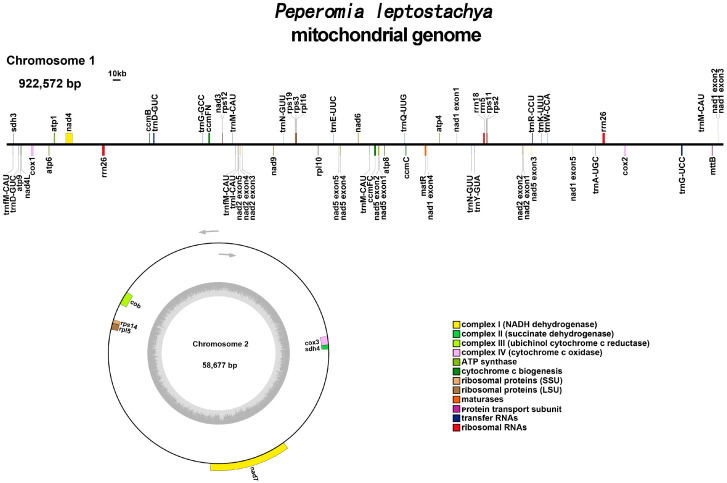
*Peperomia leptostachya* mitochondrial genome gene map. For both circular chromosomes, inner genes are transcribed clockwise and outer genes are transcribed counter-clockwise. Genes are color-coded, as indicated in the legend. This is a conventional gene map representation.

The mitochondrial genome of *P. leptostachya* is characterized by a multi-branch structure, spanning 981,249 bp with a GC content of 44.03% (Figure 1). Annotation identified a total of 52 genes, which included 35 distinct protein-coding genes, 14 tRNA genes, and 3 rRNA genes (Table S1). Among the protein-coding genes, 24 are core mitochondrial genes essential for performing core functions. These core genes encompass five ATP synthase subunits (*atp1*, *atp4*, *atp6*, *atp8*, *atp9*), nine NADH dehydrogenase subunits (*nad1*, *nad2*, *nad3*, *nad4*, *nad4L*, *nad5*, *nad6*, *nad7*, *nad9*), four cytochrome C biogenesis components (*ccmB*, *ccmC*, *ccmFC*, *ccmFN*), three cytochrome C oxidase subunits (*cox1*, *cox2*, *cox3*), and one gene each for a membrane transport protein (*mttB*), a maturation enzyme (*matR*), and ubiquinol-cytochrome C reductase (*cob*). The remaining 11 protein-coding genes are non-core, consisting of three ribosomal large subunit genes (*rpl2*, *rpl5*, *rpl16*), six ribosomal small subunit genes (*rps2*, *rps3*, *rps11*, *rps12*, *rps14*, *rps19*), and two succinate dehydrogenase genes (*sdh3*, *sdh4*).

**Figure 2:**
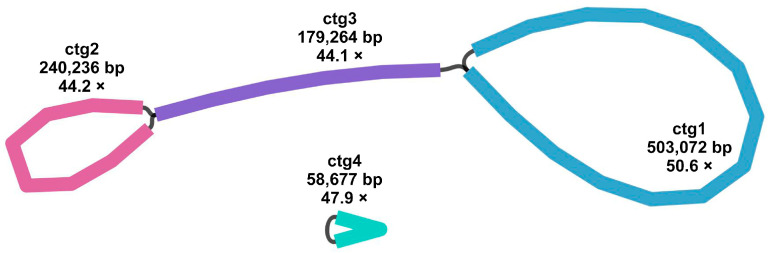
Branched conformation of *P. leptostachya* mitogenome. The figure illustrates the inferred multi-branched conformation supported by long-read evidence. Colored segments were named contig/R 1-4.

The final result is shown in Figure 2, which contains four contig sequences that formed a complex meshwork. Among them, ctg4 was a self-forming sequence, and the remaining ctg1-ctg3-ctg2 could be processed as a separate assembly unit. To ensure the reliability of this multi-branch structure, we further conducted high-fidelity long-read cross-validation. The original long-read data underwent quality filtering (Q ≥ 10, length ≥ 5 kb). High-quality reads were aligned to mitochondrial contig reference sequences (ctg1-ctg4) using BLASTn. Reliable results with alignment consistency ≥ 90% and alignment length ≥ 1 kb were selected to quantify the long-read support counts for different potential connection paths (Tables S2 and S3). Validation data revealed ctg1-ctg3-ctg2 as the dominant pathway in the multi-branch structure: the first branch node ctg2 (+)-ctg3 (+) received support from 35 long reads, while the subsequent node ctg3 (+)-ctg1 (+) received support from 15 long reads. In contrast, alternative potential paths (e.g., ctg3 (−)-ctg2 (+), ctg1 (+)-ctg1 (+)) received only two and eleven long read supports, respectively, significantly lower than the core path. These paths failed to form viable competing structures, effectively resolving structural ambiguity. Additionally, ctg4 formed an independent branch via a self-loop configuration, supported by 11 long reads. All branch nodes above received direct evidence of long-read spanning support. Combined with the analysis of pathway support differences, this further confirms the authenticity and uniqueness of the multi-branched structure of *P. leptostachya* mitochondria.

### 3.2. Codon Preference Analysis

**Figure 3:**
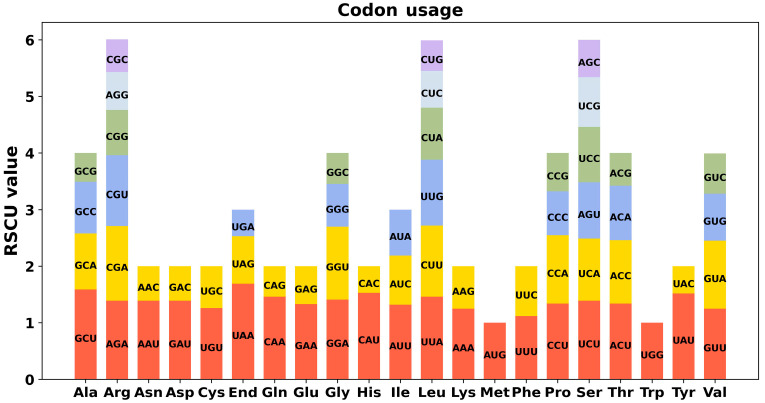
The relative synonymous codon usage (RSCU) in the *P. leptostachya* mitogenome. The x-axis represents the type of amino acid. The y-axis represents the RSCU value. Each amino acid is encoded by multiple codons, which is shown by the histogram with different colors.

Codon preference was evaluated for 35 unique PCGs of *P. leptostachya* mitochondria (Table S4). RSCU exceeding 1 typically indicated a stronger preference for the selective use of specific amino acids. Figure 3 shows that, except for AUG and UGG with RSCU values of 1, PCGs also exhibit widespread codon bias. UAA leads all PCGs with the top RSCU value at 1.69.

### 3.3. Repeat Sequences of Mitochondria

**Figure 4:**
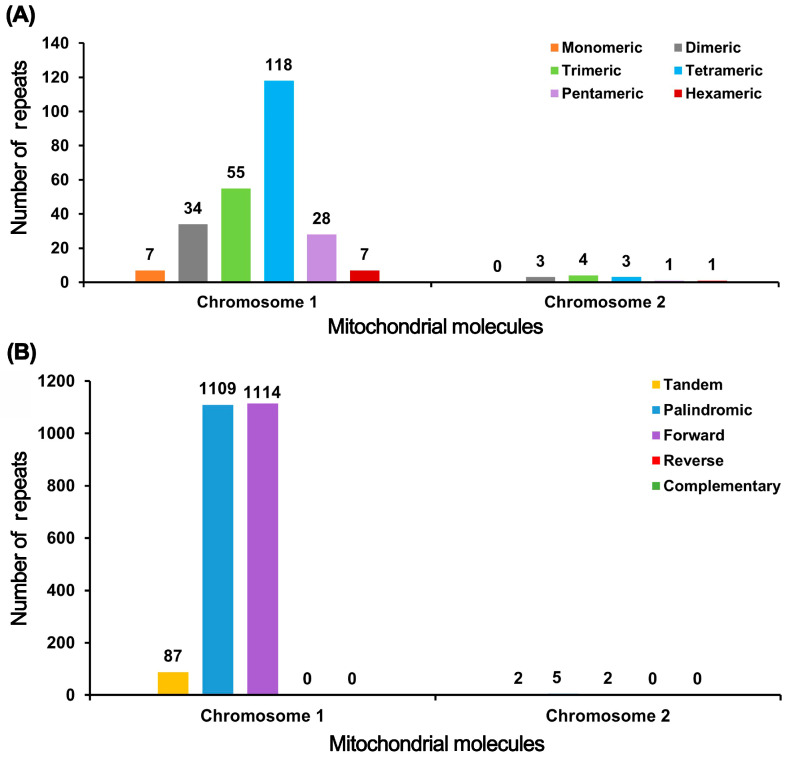
Repeat sequences analysis histogram. (**A**) The horizontal axis represents mitochondrial molecules; the vertical axis represents the number of repeat fragments. The orange legend indicates monomeric SSRs, the gray legend indicates dimeric SSRs, the green legend indicates trimeric SSRs, the blue legend indicates tetrameric SSRs, the purple legend indicates pentameric SSRs, and the red legend indicates hexameric SSRs. (**B**) The horizontal axis represents mitochondrial molecules, the vertical axis represents the number of repeat fragments, the yellow legend indicates tandem repeats, the blue legend indicates palindromic repeats, the purple legend indicates forward repeats, the red legend indicates reverse repeats, and the green legend indicates complementary repeats.

Chromosome 1 contained 249 SSRs, and the tetramer-type SSRs made up 47.39% of all identified SSRs (Figure 4). There were 87 tandem repeats in Chromosome 1 with a 65% or higher match rate and sizes varying between 7 and 127 bp. The scattered repeats were detected. There were 2223 sequences (>50 bp), which included 1109 pairs of palindromic repeats and 1114 pairs of forward repeats. No reverse or complementary repeats were discovered in the analysis. Among these sequences, the longest palindromic repeat stretched to 13,288 bp, and the longest forward repeat reached 4080 bp.

Chromosome 2 contained 12 SSRs, and no monomer SSRs were detected (Figure 4). Chromosome 2 contains two tandem repeats with matching degrees of 92% and 96%, respectively, and measuring 22 bp and 23 bp, respectively. The scattered repeat sequences in Chromosome 2 were detected. A total of seven repeat pairs with lengths ≥ 50 bp were found, comprising five palindromic repeats and two forward repeats. The maximum palindromic sequence measures 169 bp, while the maximum direct repeat spans 93 bp.

A total of 225 SSRs were identified in the closely related species *P. nigrum*. Monomeric SSRs were the most abundant, accounting for 34.22% (77 SSRs) of the total. Hexameric SSRs were the least common, representing only 1.33% (3 SSRs). *P. nigrum* contained 36 tandem repeat sequences with a match similarity greater than 75% and lengths ranging from 12 to 63 bp. Scattered repeat sequences were detected in *P. nigrum*, revealing 596 pairs of repeats with lengths ≥ 50 bp. Among these, 271 pairs were palindromic repeats and 325 pairs were forward repeats. No reverse repeats or complementary repeats were detected. The longest palindromic repeat measured 44,129 bp, while the longest forward repeat reached 59,435 bp.

The mitochondrial genomes of *P. leptostachya* and *P. nigrum* exhibit striking contrasts in repetitive sequence characteristics. *P. leptostachya* possesses a longer (0.98 Mb) genome with higher repetitive sequence density, featuring a significantly greater number (2230 pairs) and density (2275.51 pairs/Mb) of scattered repetitive sequences compared to *P. nigrum*. Its SSRs are predominantly tetramers. In contrast, although *P. nigrum* possesses a smaller genome (0.53 Mb) and fewer total repetitive sequences, it exhibits a higher density of SSRs per unit length (Table S5). This indicates differences in genomic rearrangement and recombination capacity between the two related species, which may influence the evolution and stability of their mitochondrial genomes.

### 3.4. Chloroplast to Mitochondrion DNA Transformation

**Figure 5:**
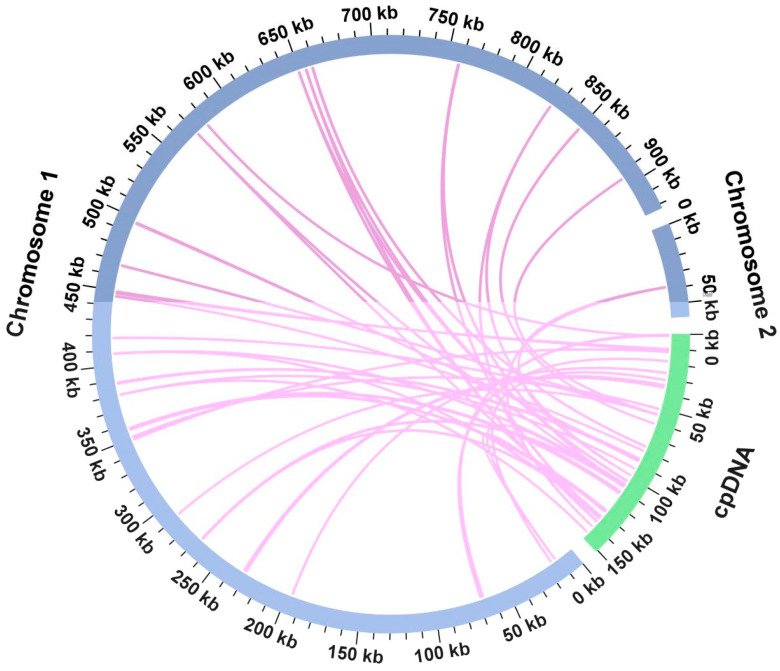
Sequence migration analysis. The purple arcs in the figure represent the mitochondrial genome, while the green arcs represent the chloroplast genome. The pink lines connecting the arcs correspond to homologous genomic segments.

Figure 5 shows that, it was found that 34 *P. leptostachya* segments exhibited homology with corresponding sections in both the mitochondrial and chloroplast genomes, spanning 9246 bp (0.94% of the genome’s total sequence). MTPT1 is the longest at 1592 bp. Three complete genes, including *psbN*, *rpl22*, and *trnD-GUC*, are present across 34 homologous fragments.

### 3.5. Phylogenetic Relationships

**Figure 6:**
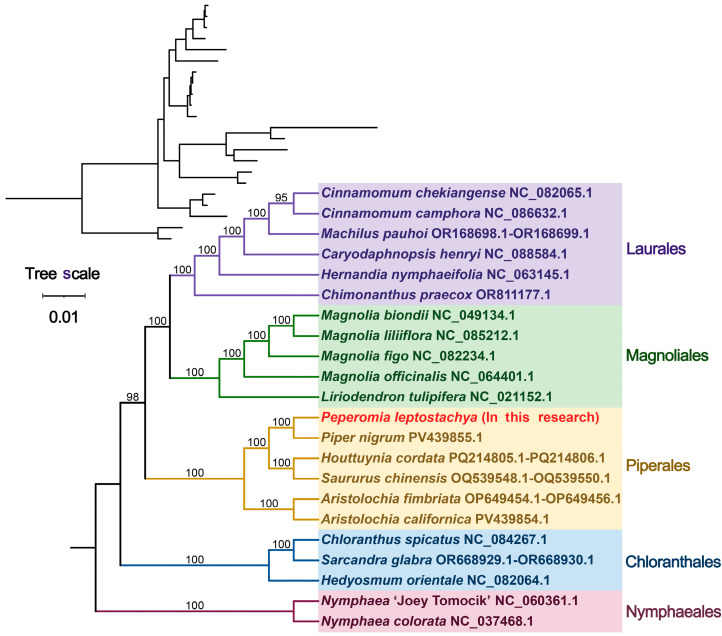
The phylogenetic relationships of *P. leptostachya* with other closely related species. Phylogenetic tree of 22 angiosperms based on the sequences of 33 conserved mitochondrial PCGs. Two Nymphaeales species was chosen as the outgroup. The number at each node is the bootstrap probability.

By analyzing mitochondrial DNA sequences from 33 conserved protein-coding genes across 22 angiosperm species in five orders, we constructed comprehensive phylogenetic trees (Figure 6). To establish an external reference point, mitochondrial genomes from two Nymphaeales species were designated as extragroups. The phylogenetic topologica structure derived from mitochondrial DNA data perfectly aligned with the APG classification. *P. leptostachya* is closely related to *P. nigrum.*

### 3.6. RNA Editing Event Analysis

**Figure 7:**
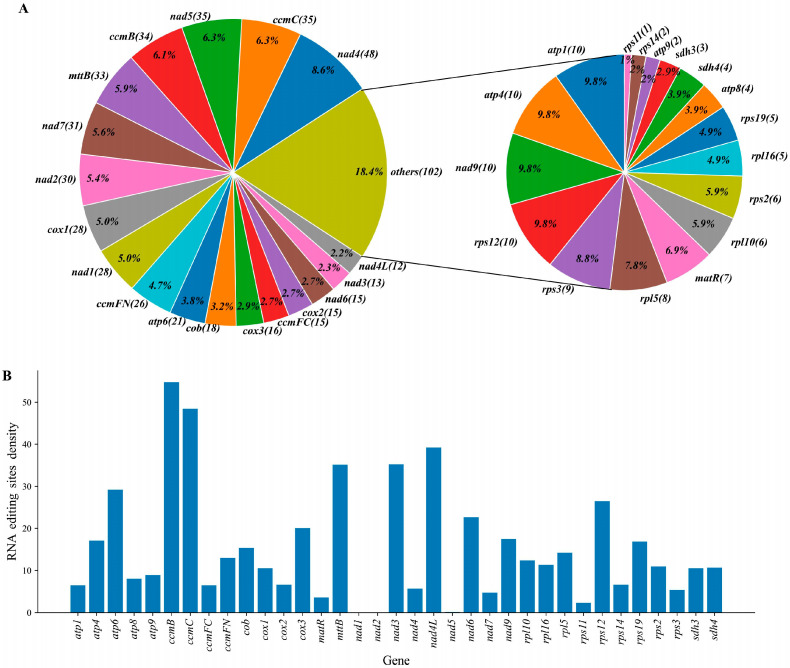
Distribution and editing site density of RNA editing sites in *P. leptostachya* mitochondrial genes. (**A**) Distribution of RNA editing sites in *P. leptostachya* mitochondrial genes. (**B**) RNA editing site density in *P. leptostachya* mitochondrial genes. RNA editing site density was defined as the number of RNA editing sites detected in each gene, normalized by the gene length (in base pairs, bp) and then scaled by a factor of 1000 to yield the number of editing sites per kilobase (sites/kb).

We detected RNA editing occurrences across 35 distinct mitochondrial protein-coding genes in *P. leptostachya* by establishing a strict threshold value of 0.9. Applying this benchmark, we uncovered 555 potential RNA editing locations spanning all 35 mitochondrial PCGs. All were cytosine-to-uracil base edits. The nad4 gene contained 48 sites for RNA editing, exhibiting the highest editing frequency at 8.6%. Secondly, the *ccmC* and nad5 genes were both identified with 35 RNA editing sites, accounting for 6.3% and 6.1% respectively (Figure 7A).

To eliminate the confounding effect of gene length on editing events, the number of editing sites was normalized by dividing it by the length of each genome. *ccmB* and *ccmC* exhibited the two highest editing site densities, reaching up to 54/kb and 48/kb, respectively (Figure 7B). These data indicate that *ccmB* and *ccmC* are more susceptible to editing at the mRNA level than other genes.

### 3.7. Collinearity Analysis

**Figure 8:**
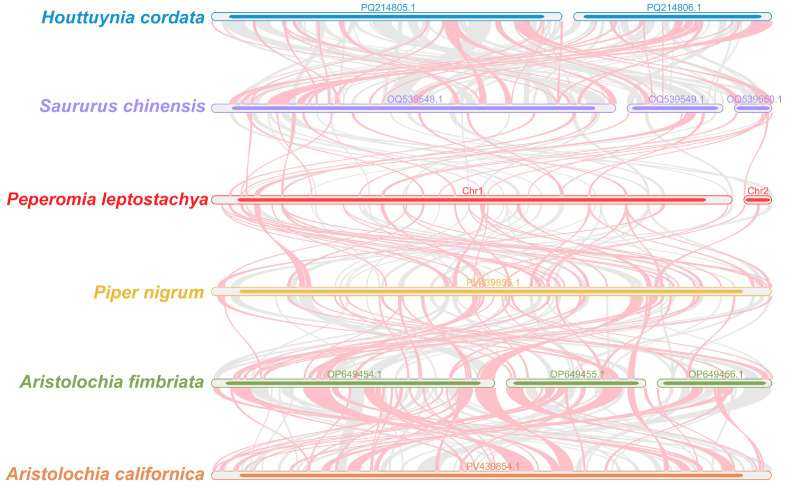
Collinearity analysis. The bars indicate the mitochondrial genomes, and the ribbons show the homologous sequences between adjacent species. The red areas indicate where the inversion occurred, and the gray areas indicate regions of high homology. The regions with no colinear blocks are indicated as unique in the species.

Illustrated in Figure 8, the red arc-shaped regions represent the inverted segments, whereas the grays denoted regions of significant similarity. Colinear blocks shorter than 0.5 kb in length were omitted from findings. We defined the threshold for collinear blocks as follows: only results with identity greater than 70% and alignment length exceeding 500 bp were retained for subsequent analysis. To quantify the degree of genomic rearrangement, we primarily compared the number and size of collinear blocks. Although collinear blocks homologous between *P. leptostachya* and its congeners were detected, these blocks exhibited a relatively brief length. Additionally, unidentified areas appeared. A total of 571 colinear blocks were detected among these six species. *P. leptostachya* and *Saururus chinensis* shared 71 colinear blocks with a combined length of 77,769 bp, the longest being 3007 bp. *P. leptostachya* and the congeneric species *P. nigrum* shared 109 colinear blocks with a total length of 128,501 bp, the longest being 3021 bp. These results indicate that *P. leptostachya* shares more colinear blocks with *P. nigrum*, a species within the same family, while the number and length of colinear blocks decrease in species with more distant phylogenetic relationships. The mitochondrial genomes of the six species displayed divergent collinear block arrangements. The *P. leptostachya* mitochondrial genome underwent structural reorganization with that of its closely related species.

## 4. Discussion

Mitochondria are essential for numerous metabolic functions, achieving this by converting organic substances into chemical energy to power the cell’s daily operations [46,47,48]. On the other hand, mitochondria are semi-autonomous organelles, and although plant mitochondrial DNA is usually assembled into circular maps, it appears that plant mitochondrial DNA is not structured as one big circular molecule, but rather forms a complicated and ever-changing assortment of linear DNA segments [49,50,51,52]. The *P. leptostachya* mitochondrial genome was assembled from sequencing data, with a size of 981,249 bp. The published mitochondrial genome size of *Aristolochia fimbriata* in the Piperales order is 349,849 bp [53], and that of *Saururus chinensis* is 157,063 bp [54]. GC levels significantly impact species assessment [55]. The GC content of the mitochondrial genome of *P. leptostachya* is 44.03%, close to that of *Saururus chinensis* (46.4%). The mitochondrial genome of *P. leptostachya* contains four contig sequences, forming a complex network structure. Among them, ctg4 is a self-circular sequence, and the remaining ctg1-ctg3-ctg2 can be treated as a single assembly unit.

Currently, several tools are available for assembling plant mitochondrial genomes, such as PMAT, HiMT, and OATK [19]. These tools typically employ an “enrichment first, assembly later” strategy. The advantage of this approach lies in its ability to significantly reduce computational resource consumption, as it assembles only the target data. However, a potential drawback is incomplete assembly, where less conserved mitochondrial regions may be omitted during enrichment, resulting in final assemblies containing only partial genomic sequences. In contrast, our method employs a “assemble first, then filter” strategy. We performed de novo assembly of all sequencing data using Flye, yielding a mixed assembly containing nuclear, chloroplast, and mitochondrial genomes. Subsequently, we aligned the entire assembly against mitochondrial gene sequences from closely related species as query sequences to extract mitochondrial-related sequences. In subsequent steps, these extracted sequences served as reference sequences. Reads from the raw sequencing data that map to these reference sequences were selected for a subsequent round of mixed assembly, further enhancing assembly quality. In summary, the fundamental difference between existing tools and our approach lies in the sequence of data processing. Our approach ensures that all sequencing data are utilized for assembly, maximizing genomic sequence completeness. However, this strategy incurs significant computational resource demands and yields assembly results containing substantial interference sequences from the nuclear and chloroplast genomes. Additionally, the subsequent manual screening of mitochondrial sequences from vast datasets is cumbersome and lacks standardized procedures.

The eukaryotic genome has 64 codons, and notable variations exist in the codon utilization frequency across different plant genomes. This preference is believed to have gradually evolved into a comparative stability in cells through evolutionary selection. UAA leads all PCGs with the top RSCU value at 1.69.

Research indicates the necessity of repetitive sequences in molecular remodeling processes [56]. This study found that Chromosome 1 exhibited an extremely high abundance of repetitive sequences, with a much greater number of SSRs (249, nearly half of which were tetramers), tandem repeats (87), and large interspersed repeats (2223 pairs, mainly palindromic and forward repeats) compared to Chromosome 2 (12 SSRs, 2 tandem repeats, and 7 pairs of interspersed repeats). No inverted or complementary repeats were detected on either chromosome. Since such repetitive sequences readily form secondary structures that may lead to genomic instability, their presence or distribution within the genome is constrained by stability considerations during evolution. Chromosome 1 contained extremely long palindromic repeats (13,288 bp) and forward repeats (4080 bp), while the longest repeats on Chromosome 2 were much shorter (<170 bp), further highlighting the fundamental differences between the two chromosomes in terms of repetitive sequence scale, complexity, and possible evolutionary paths or functional constraints. This study reveals significant differences in the composition, distribution, and density of repetitive sequences between the mitochondrial genomes of two Piper species. *P. leptostachya*, possessing a longer chromosome (approximately 0.98 Mb), exhibits a higher number and density of scattered repetitive sequences, predominantly palindromic repeats and forward repeats, with tetrameric SSRs being the dominant form. In contrast, the smaller genome of *P. nigrum* (approximately 0.53 Mb) contains fewer total repetitive sequences but exhibits higher SSR density per unit length, with monomeric SSRs as the predominant type. These differences may reflect distinct adaptive strategies in genomic stability, repetitive sequences, and evolutionary history between the two species. The longest forward repeat identified in *P. nigrum* spans 59,435 bp, potentially linked to genomic rearrangements or specific functional regulation. Future studies integrating transcriptomic data could further elucidate the precise roles of these repeats in gene expression regulation and genomic evolution.

During the evolution of mitochondria, chloroplast fragments can integrate into the mitochondrial genome [57,58]. Thirty-four mitochondrial plastid DNAs (MTPTs) were identified in *P. leptostachya*, representing 0.94% of the mitochondrial genome sequence. The largest fragment (MTPT1) amounted to 1592 bp. Importantly, these fragments retained three complete functional genes (*psbN*, *rpl22*, and *trnD-GUC*). These findings suggest that during evolution, the mitochondria of *P. leptostachya* may have not only incorporated DNA sequences of chloroplast origin but also retained and maintained the integrity of some of these genes to a certain extent. This phenomenon provides evidence for the potential role of inter-organelle gene transfer in shaping the composition of mitochondrial genomes.

Five hundred and fifty-five high-confidence RNA editing sites were detected, with all observed edits corresponding to the C→U type. These corroborated the typical features of plant mitochondrial RNA editing. The respiratory chain complex I core subunit genes nad4 and *nad5*, as well as cytochrome c biosynthesis key genes, exhibited significant editing site enrichment. High-frequency RNA editing may therefore serve as a post-transcriptional regulatory mechanism, potentially exhibiting associations with genes related to energy metabolism. This may involve correcting non-optimal codons, regulating translation efficiency, or maintaining protein structural stability.

The identification of RNA editing sites in this study relies on bioinformatics prediction, and the results carry inherent uncertainty primarily manifested in two aspects: (1) Impact of cutoff threshold selection: The editing site threshold of 0.9 employed in this study effectively filters low-confidence sites. However, adjusting this threshold may cause fluctuations in site count and density. Future studies could systematically evaluate threshold effects by implementing gradient thresholds. (2) Limitations due to lack of experimental validation and cross-species comparison: The 555 high-confidence sites identified in this study have not been validated via RT-PCR or other molecular biology experiments, and some predicted sites may be false positives. Additionally, due to the current scarcity of mitochondrial RNA editing data from closely related species, cross-species comparative analysis is not feasible at present, making it difficult to determine the evolutionary conservation of these editing sites. Future studies will supplement experimental validation and expand species sampling to further explore the functional and evolutionary significance of mitochondrial RNA editing sites in *P. leptostachya*.

Co-linearity analysis reveals significant structural divergence. This is characterized by shortened breaks in the covariate block, the appearance of unique sequence regions, and overall extreme non-conservatism. Together, these features suggest that *P. leptostachya* has undergone a mitochondrial genome rearrangement, and that the sequences unique to *P. leptostachya* may originate from rapidly diverging non-coding regions, horizontally shifted genes, or amplification of species-specific repetitive sequences. It is suggested that the mitochondrial genome of *P. leptostachya* has undergone dramatic structural remodeling, resulting in a highly inconsistent arrangement of common lineage blocks between *P. leptostachya* and closely related species.

The phylogenetic tree generated exhibited a topological configuration that closely aligned with the APG classification system for angiosperms, which supported the utility of mitochondrial genomic data as an effective tool for reconstructing angiosperm evolutionary relationships. We revealed that *P. leptostachya* is closely related to P. nigrum, which corroborates the phylogenetic attribution of the species by traditional morphological classification. Whether the mitochondrial genome characteristics and RNA editing patterns observed in *P. leptostachya* in this study are widespread within the genus Piper and even the family Piperaceae requires validation through the following systematic work. Sampling must be expanded to include diverse geographic populations of *P. leptostachya*, representative species within the genus Peperomia, and other key genera within the Piperaceae family. Comparative genomics analysis should then be conducted to clarify the conservation and divergence patterns of structural features and RNA editing patterns. Concurrently, phylogenetic analysis incorporating chloroplast genome data should be performed for cross-validation. Only through such multidimensional, cross-taxon systematic research can the taxonomic applicability of these features be accurately defined.

Post-transcriptional regulation of organelles serves as a pivotal link between genetic information and biological functions. Editing mechanisms not only repair protein functional defects but also fine-tune protein activity, thereby enhancing environmental adaptability and survival advantages. Post-transcriptional regulation of organelles—particularly RNA editing—holds profound biological significance. Paschalidis et al. provided supporting evidence for this theory through their study of the medicinal plant *Sideritis syriaca* subsp. *syriaca* [18]. They identified species based on conserved organelle DNA sequences and examined agronomic practices that influence organelle metabolic output.

The mitochondrial genome of *P. leptostachya* revealed in this study exhibits distinctive characteristics and significant application value. Based on its unique sequences, high-frequency RNA editing sites, and functional gene features, highly specific molecular markers can be developed for the precise identification and quality control of *P. leptostachya* medicinal materials. This mitochondrial genome serves as a reliable molecular marker for angiosperm phylogenetic reconstruction, clarifying the phylogenetic relationships between *P. leptostachya* and its closely related species. It provides a genomic tool for germplasm resource classification, identification, and conservation within the Piperaceae family. The identified high-frequency RNA editing sites in mitochondrial-plastid transfer genes (MTPTs) and energy metabolism genes not only reveal the roles of organelle-to-organelle gene transfer and post-transcriptional regulation in genomic dynamic evolution but also provide new clues for exploring plant energy metabolism regulation mechanisms and potential biotechnological applications. These findings offer valuable references for genetic breeding and bioprospecting efforts in Piperaceae plants.

## 5. Conclusions

The mitochondrial genome of *P. leptostachya* has been assembled, annotated, and subjected to gene sequence analysis. This study reports the first mitochondrial genome of *P. leptostachya*, marking the first mitochondrial genome published within the genus Peperomia and the second within the Piperaceae family. The genome exhibits significant structural peculiarities, presenting as an atypical circular structure accompanied by an independent minicircle, forming a multi-branched reticulate configuration spanning a total length of 981,249 bp. The two chromosomes show marked differences in repetitive sequence composition, suggesting potential divergence in their evolutionary pathways and functional constraints, reflecting the dynamic and complex nature of mitochondrial genome structure. The study identified 34 mitochondrial transfer genes (MTPTs) within the genome, accounting for 0.94% of its total length. These retain three fully functional genes, confirming the dynamic nature of inter-organelle gene transfer. Phylogenetic analysis based on 33 conserved mitochondrial genes provides evidence for the classification of *P. leptostachya* and its phylogenetic relationship with P. nigrum. These findings are consistent with morphological classification and existing molecular phylogenetic frameworks. This study provides molecular evidence for the genetic characteristics and evolutionary pathways of *P. leptostachya*, laying the foundation for research into its biological traits and molecular breeding.

## Data Availability

The genome sequence data are openly available in GenBank of the NCBI at https://www.ncbi.nlm.nih.gov/ (accessed on 16 October 2024) under accession Nos. PQ468038 and PQ468039.

## Supplementary Materials

The following supporting information can be downloaded at: https://www.mdpi.com/article/10.3390/genes17010118/s1, Table S1: Genes encoded in the mitochondrial genome of *Peperomia leptostachya*; Table S2: Potential path and number of long reads; Table S3: Collection of long reads obtained from BLASTn results supporting different paths; Table S4: Relative synonymy usage of codons for each amino acid pair; Table S5: Comparison of the number and density of mitochondrial genome duplicate sequences in *P. leptostachya* and *Piper nigrum*.
