# Supplementary material for: Complete Mitochondrial Genomic Characteristics and Phylogenetic Analysis of the Medicinal Plant Peperomia leptostachya
Source: Genes (Basel). 2026 Jan 22;17(1):118. doi: 10.3390/genes17010118 (PMC12841264; doi:10.3390/genes17010118)
Supplement: Supplementary file 1 [file genes-17-00118-s001.zip › genes-4081837-supplementary.pdf]

Table S1. Genes encoded in the mitochondrial genome of *Peperomia leptostachya*

Group of genes	Name of genes
ATP synthase	atp1，atp4，atp6，atp8，atp9
NADH dehydrogenase	nad1，nad2，nad3，nad4，nad4L，nad5，nad6，nad7，nad9
Cytochrome b	cob
Cytochrome c biogenesis	ccmB，ccmC，ccmFC，ccmFN
Cytochrome c oxidase	cox1，cox2，cox3
Maturases	matR
Protein transport subunit	mttB
Ribosomal protein large subunit	rpl5，rpl10，rpl16
Ribosomal protein small subunit	rps2，rps3，rps11，rps12，rps14，rps19
Succinate dehydrogenase	sdh3，sdh4
Ribosome RNA	rrn5，rrn18，rrn26（×2）
Transfer RNA	trnA-UGC，trnD-GUC（×2），trnE-UUC，trnfM-CAU（×2），trnG-GCC，trnG-UCC，trnI-CAU, trnK-UUU，trnM-CAU（×3），trnN-GUU（×2），trnQ-UUG，trnR-CCU，trnW-CCA，trnY-GUA

Table S4. Relative synonymy usage of codons for each amino acid pair

Amino	Codon 1 RSCU	Codon 2 RSCU	Codon 3 RSCU	Codon 4 RSCU	Codon 5 RSCU	Codon 6 RSCU
Ala	GCU	GCA	GCC	GCG		
	1.59	0.99	0.91	0.51		
Arg	AGA	CGA	CGU	CGG	AGG	CGC
	1.39	1.32	1.25	0.8	0.67	0.58
Asn	AAU	AAC				
	1.39	0.61				
Asp	GAU	GAC				
	1.39	0.61				
Cys	UGU	UGC				
	1.26	0.74				
End	UAA	UAG	UGA			
	1.69	0.84	0.47			
Gln	CAA	CAG				
	1.46	0.54				
Glu	GAA	GAG				
	1.33	0.67				
Gly	GGA	GGU	GGG	GGC		
	1.41	1.29	0.75	0.55		
His	CAU	CAC				
	1.53	0.47				
Ile	AUU	AUC	AUA			
	1.32	0.87	0.81			
Leu	UUA	CUU	UUG	CUA	CUC	CUG
	1.46	1.26	1.16	0.92	0.65	0.54
Lys	AAA	AAG				
	1.25	0.75				
Met	AUG					
	1.0					
Phe	UUU	UUC				
	1.12	0.88				
Pro	CCU	CCA	CCC	CCG		
	1.34	1.21	0.77	0.68		
Ser	UCU	UCA	AGU	UCC	UCG	AGC
	1.39	1.1	0.99	0.98	0.88	0.66
Thr	ACU	ACC	ACA	ACG		
	1.34	1.12	0.96	0.58		
Trp	UGG					
	1.0					
Tyr	UAU	UAC				
	1.52	0.48				
Val	GUU	GUA	GUG	GUC		
	1.25	1.2	0.83	0.71		

Table S5. Comparison of the Number and Density of Mitochondrial Genome Duplicate Sequences in *Peperomia leptostachya* and *Piper nigrum*

species	Mitochondrial genome length（Mb）	SSRs		Tandem repeats		Dispersed repeats	
		Number	Number of repetitive sequences per mb	Number	Number of repetitive sequences per mb	Number	Number of repetitive sequences per mb
Peperomia leptostachya	0.98	261	266.33	89	90.82	2230	2275.51
Piper nigrum	0.53	225	424.53	36	67.92	596	1124.53
